# DeepNup: Prediction of Nucleosome Positioning from DNA Sequences Using Deep Neural Network

**DOI:** 10.3390/genes13111983

**Published:** 2022-10-30

**Authors:** Yiting Zhou, Tingfang Wu, Yelu Jiang, Yan Li, Kailong Li, Lijun Quan, Qiang Lyu

**Affiliations:** 1 School of Computer Science and Technology, Soochow University, Suzhou Ganjiang East Streat 333, Suzhou 215006, China; 2 Key Lab for Information Processing Technologies, Soochow University, Suzhou Ganjiang East Streat 333, Suzhou 215006, China; 3 Collaborative Innovation Center of Novel Software Technology and Industrialization, Organization, Nanjing 210000, China

**Keywords:** deep learning, bioinformatics, nucleosome positioning, multiscale convolution neural network, gated recurrent unit

## Abstract

Nucleosome positioning is involved in diverse cellular biological processes by regulating the accessibility of DNA sequences to DNA-binding proteins and plays a vital role. Previous studies have manifested that the intrinsic preference of nucleosomes for DNA sequences may play a dominant role in nucleosome positioning. As a consequence, it is nontrivial to develop computational methods only based on DNA sequence information to accurately identify nucleosome positioning, and thus intend to verify the contribution of DNA sequences responsible for nucleosome positioning. In this work, we propose a new deep learning-based method, named DeepNup, which enables us to improve the prediction of nucleosome positioning only from DNA sequences. Specifically, we first use a hybrid feature encoding scheme that combines One-hot encoding and Trinucleotide composition encoding to encode raw DNA sequences; afterwards, we employ multiscale convolutional neural network modules that consist of two parallel convolution kernels with different sizes and gated recurrent units to effectively learn the local and global correlation feature representations; lastly, we use a fully connected layer and a sigmoid unit serving as a classifier to integrate these learned high-order feature representations and generate the final prediction outcomes. By comparing the experimental evaluation metrics on two benchmark nucleosome positioning datasets, DeepNup achieves a better performance for nucleosome positioning prediction than that of several state-of-the-art methods. These results demonstrate that DeepNup is a powerful deep learning-based tool that enables one to accurately identify potential nucleosome sequences.

## 1. Introduction

Nucleosome positioning generally refers to the precise location of nucleosomes on the genomic DNA sequence [1]. Nucleosomes are the fundamental structural units of eukaryotic chromatin, which are a 147 base pairs (bp) segment of DNA wrapped around a histone octamer by approximately 1.65 turns [2,3], referred to as core DNA or nucleosomal DNA. Nucleosomes are connected to form a structure resembling beads on a string by a short segment of linker DNA [4], the length of which varies from 20 to 54 bp. Each histone octamer consists of two copies each of the highly conserved histones H2A, H2B, H3, and H4, and the linker histone H1 serves to stabilize the nucleosome structure [5].

In eukaryotic cells, nucleosome positioning has been implicated to be critical in a diversity of biological processes [6,7,8,9], for instance, the precise location of nucleosomes enables the regulation of the genomic sequence accessibility to DNA-binding proteins, so as to achieve the regulation of gene expression [10,11], DNA replication [12,13,14], and repair [15,16]. Consequently, accurately identifying the precise location of nucleosomes along the DNA sequences may deepen the understanding of diverse biological processes. Nucleosome positioning is a complex process involving interactions between many factors, including DNA sequences, transcription factors, histone modifying enzymes, and chromatin remodeling compounds [1,17,18], but which of these factors are the major determinants remain unknown. Previous studies indicated that the intrinsic preference of nucleosomes for DNA sequences may be a dominant determinant in nucleosome positioning [19,20], for instance, some research reported that approximately 75% of nucleosomes are characterized by DNA sequences [21]. As a consequence, it is nontrivial to develop computational methods based on DNA sequence information for identifying nucleosome positioning.

Thanks to the development of high-throughput experimental techniques, covering DNase I hypersensitive sites sequencing (DNase-seq) [22], micrococcal nuclease sequencing (MNase-seq) [23], and chromatin immunoprecipitation sequencing (ChIP-seq) [7], high-resolution maps of nucleosomes have been obtained for several species, such as yeast [24], *Homo sapiens* (*H. sapiens*) [25], *Caenorhabditis elegans* (*C. elegans*) [26], and *Drosophila melanogaster* (*D. melanogaster*) [27]. These high-resolution data have greatly facilitated the development of various computational methods to make an accurate prediction of nucleosome positioning.

To date, a variety of computational methods have been proposed to predict nucleosome positioning [28,29,30]. For example, several biophysical methods based on the deformation energy [31] or thermodynamic stability [32] of DNA sequences have emerged to predict nucleosome positioning. Additionally, a number of machine learning (ML)-based nucleosome positioning methods have also emerged, such as based on hidden Markov model [33] or support vector machine (SVM) [34]. The predictor, called iNuc-PseKNC [35] and based on SVM, first extracts a feature vector of pseudo k-tuple nucleotide composition incorporating six local DNA structural properties to formulate a DNA sequence sample, and then feeds the obtained feature vector into a SVM classifier to predict nucleosome positioning in *H. sapiens*, *C. elegans*, and *D. melanogaster* species. iNuc-PseKNC has been demonstrated to outperform the previously proposed predictors in terms of the prediction performance. Nonetheless, these ML-based methods require to manually extract features from DNA sequences. Fortunately, with the rapid development of deep learning (DL) technologies [36,37,38], this limitation can be alleviated by DL, which is capable of automatically learning feature representations from raw data. In recent years, several DL-based methods for nucleosome positioning prediction have been developed and have achieved a competitive performance [30,39,40]. The first DL-based nucleosome positioning predictor is called LeNup [41], which encodes DNA sequences into one-hot form as inputs, and combines Inception network with gated convolutional neural network (CNN) to learn the nucleosome positioning features from DNA sequences. By comparing several ML-based methods, LeNup achieved a better performance measured through 20-fold cross validation on the same benchmark datasets. Meanwhile, the method NucPosPred [42] was developed by combining four different feature codes, PSTNP, k-tuple nucleotide composition, KNN features, and EIIP, and uses XGBoost and SVM to classify DNA sequences. Subsequently, another DL-based method called DLNN [43] was developed by encoding raw DNA sequences into one-hot form as inputs and stacking CNN and Long Short-Term Memory (LSTM) network to extract local and long-range features of nucleosome positioning sequences. The experimental results demonstrated that DLNN-5 exhibits an excellent prediction performance via 10-fold cross validation on the 2 benchmark datasets.

Although DL-based methods have made much progress in the nucleosome positioning prediction, there is still room for improvement in the prediction performance and it deserves further research. As a result, in this work, a novel DL-based method, namely DeepNup (Deep learning for nucleosome positioning prediction), is developed to improve the prediction performance of nucleosome positioning. More specifically, a hybrid feature encoding scheme consisting of One-hot encoding and trinucleotide composition (TNC) encoding is utilized to encode DNA sequences. DeepNup uses two parallel multi-scale convolutional neural network (MCNN) modules consisting of two parallel convolution kernels of different sizes for extracting abstract and detailed local features, respectively, and uses two sequentially gated recurrent units ( GRU) to extract long-range features. Finally, a fully connected (FCNN) layer and a sigmoid unit are used as a classifier to generate the final prediction outcomes. To make a comprehensive comparison to the state-of-the-art methods, DeepNup are evaluated via 10-fold cross validation on 2 benchmark datasets of nucleosome positioning. The comparison results indicate that DeepNup exhibits a competitive performance for nucleosome positioning prediction across two benchmark datasets compared to two baseline methods. These results show that DeepNup is a powerful DL-based tool for accurately identifying potential nucleosome sequences. The source code of DeepNup is available at https://github.com/lennylv/DeepNup, accessed on 29 September 2022.

## 2. Methods

In this section, we first introduce a hybrid feature encoding scheme consisting of One-hot encoding and Trinucleotide composition encoding to encode the DNA sequences, then describe the design of our proposed DeepNup in detail, including the basic modules and their parameter settings, and finally give an introduction to the training methodology of DeepNup.

### 2.1. Hybrid Feature Encoding Scheme

DNA sequences consists of four nucleotides: ′A’ (Adenine), ′C’ (Cytosine), ′G’ (Guanine), and ′T’ (Thymine), which is not suitable as input to machine learning algorithms. Consequently, the symbolic representations of DNA sequences should be converted into a numerical form that is easy to process for machine learning algorithms. In this work, we use a hybrid feature encoding scheme based on DNA sequences, in which a DNA sequence as input is encoded by using One-hot encoding and TNC encoding [44]. The former can preserve the nucleotide composition of each position in the DNA sequences, while the latter can capture the local sequence order information in the DNA sequences.

#### 2.1.1. One-Hot Encoding

One-hot encoding converts each categorical variable into a separate feature consisting of binary values (i.e., 1 or 0). Therefore, by using One-hot encoding, each nucleotide is encoded into a four-dimensional one-hot vector, i.e., ′A’, ′C’, ′G’, and ′T’ are encoded by (1, 0, 0, 0), (0, 1, 0, 0), (0, 0, 1, 0), and (0, 0, 0, 0, 1), respectively. Following this notation, a DNA sequence of length *L* is transformed into a binary matrix of dimension L×4. In this work, by using One-hot encoding, a 147 bp DNA sequence will be encoded into a binary feature matrix of dimension 147×4.

#### 2.1.2. TNC Encoding

The *k*-tuple nucleotide composition refers to the frequencies of all possible polynucleotide with *k* nucleotides occurring in the sequence. In this work, we set k=3 indicating trinucleotide composition (TNC). The TNC encoding is defined as the occurrence frequencies of continuous trinucleotides in the predicted DNA sequence. Specifically, the four nucleotides ′A’, ′C’, ′G’, and ′T’ can form 64 trinucleotide combinations, such as ′AAA’, ′AAC’, ′AAG’, etc. For a DNA sequence of length *L*, the TNC encoding will transform the DNA sequence into a 64 dimensional vector S, wherein the vector S is defined as:

S=[f(A,A,A),f(A,A,C),…,f(T,T,T)]

where:

$$f\left(i,j,k\right)=\frac{n_{\left(i,j,k\right)}}{L-2},\;i,j,k\in \left\{A,C,G,T\right\}$$

wherein f(i,j,k) denotes the frequency of the trinucleotide of type (i,j,k) and $n_{\left(i,j,k\right)}$ denotes the number of occurrences of the trinucleotide of type (i,j,k), respectively. In this work, by using TNC encoding, a 147 bp DNA sequence will be encoded into a 64 dimensional feature vector.

### 2.2. The Model Architecture of DeepNup

**Figure 1:**
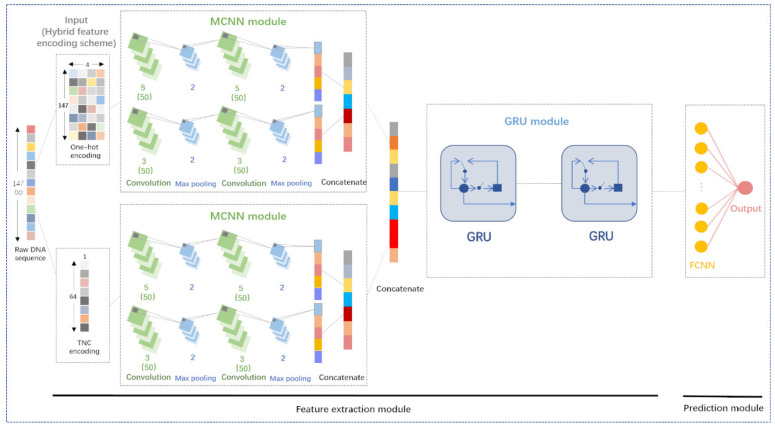
The model architecture of DeepNup. First, a raw 147 bp DNA sequence is encoded into a 147 × 4 dimensional binary matrix and a 64 dimensional vector by using One-hot encoding and TNC encoding, respectively. Next, both the resulting binary feature matrix and feature vector are simultaneously fed into the two parallel MCNN modules to extract local features of DNA sequences. As a result, two feature representations are obtained and merged into a feature representation, which is then fed into the GRU module to extract the global features of DNA sequences. Finally, a FCNN layer and a sigmoid unit are used as a binary classifier to generate a final prediction result.

In this work, we design a new DL-based model, called DeepNup, the function of which is to predict nucleosome positioning based on DNA sequences only. The architecture of DeepNup is shown in Figure 1. First, DeepNup exploits the feature extraction module, which is mainly composed of an input layer, two MCNN modules, and a GRU module, to extract local and global features from the DNA sequences. Subsequently, DeepNup exploits the prediction module, which consists of a FCNN layer and an output layer, to predict the nucleosome positioning. Specifically, each DNA sequence is encoded by using One-hot encoding and TNC encoding, respectively. Next, both the resulting two feature matrices are simultaneously fed into the two parallel MCNN modules to extract more abstract and deeper local features of DNA sequences, and all these local correlation features are then concatenated and fed into the GRU module to extract global correlation features of DNA sequences. Finally, the feature matrix generated by the feature extraction module is fed into the FCNN layer and the output layer to generate the final prediction result, i.e., to predict whether the DNA sequence is a nucleosome sequence or a linker sequence. In the following, we will describe in detail the basic modules used to construct the DeepNup model and their parameter settings.

#### 2.2.1. MCNN

The structure of MCNN module is depicted in Figure 1. The MCNN module has two parallel channels, and each channel mainly involves two alternating convolution and max pooling layers with different kernel sizes. Consequently, these two channels have different receptive fields, and they are capable of capturing the features of DNA sequences in different scales. Specifically, each shallow feature representation of DNA sequences is input to the two channels of the MCNN module in parallel. As neurons in the convolution layers of the first channel have relatively large receptive fields, the first channel could detect more abstract local information of DNA sequences, while neurons in the convolution layers of the second channel have relatively small receptive fields. The second channel could detect more detailed local information of DNA sequences. The higher abstract local feature representations and the more detailed local feature representations extracted by the two channels are merged by a concatenate operator.

A one-dimensional DNA sequence is usually transformed into a two-dimensional feature matrix, and the width and depth of the input matrix correspond to the number of rows and columns of the feature matrix, respectively. Different from a three-dimensional feature tensor of an image, there is no height of a two-dimensional feature matrix of a DNA sequence. Figure 1 illustrates the hyperparameters for each convolution layer and pooling layer, including the number of kernels, the kernel size, and the pooling stride. To be specific, the notation ′*m*’ (e.g., 5, 3) beneath a convolution block means that the width of each kernel is set to m, whereas the depth of the kernels is not marked here, which depends on the number of kernels used in the previous layer. The notation ′(50)’ beneath the notation ′m’ means that there are 50 kernels in each of eight convolution blocks. In this way, the depth of the output of these convolution layers will be 50. Moreover, in each convolution layer, the output keeps the same width dimension as that of the input, each neuron exploits ReLU as the activation function, and the L2 regularization is set to 1 × 10${}^{-3}$.

Generally, a pooling layer follows a convolution layer so as to reduce the complexity of the output and prevent overfitting, which consists of max pooling and average pooling. In this work, the max pooling layer is selected. The notation ′2’ beneath each max pooling layer means that the pooling stride is set to 2. Therefore, the width dimension of the output of this layer is only half of the width dimension of the input.

Taking an input 147 × 4 feature encoding matrix as an example, each feature representation is characterized by a 36 × 50 matrix following each of the two channels in the MCNN module, then a concatenate operator is used to concatenate the two 36 × 50 matrices into a 72 × 50 matrix. The same operation is also used for an input 64 × 1 feature encoding matrix and finally generates a 36 × 50 matrix.

#### 2.2.2. GRU Module

It is generally assumed that a fragment of a DNA sequence is associated with both the sequence fragments before and after it, so the long-range dependencies of a DNA sequence should also be considered. In this work, we design a GRU module consisting of two sequential GRU layers to capture the global correlation features from DNA sequences, the structure of which is shown Figure 1.

GRU is an improved variant of LSTM, which is also capable of solving long-range dependency problems during the training of long sequences. Specifically, LSTM consists of three types of gates: input gate, output gate, and forget gate [45], whereas GRU is comprised of two types of gates, i.e., update gate and reset gate [46], wherein the function of the former is to control how much of the previous information needs to be transferred to the current state, and the function of the latter is to control how much of the previously irrelevant information is to be filtered out. Compared to LSTM, which consists of three types of gates, GRU contains fewer parameters that are needed to be learned, so a GRU network will have a more efficient training performance than a LSTM network.

Based on previous studies, a widely adopted strategy for taking advantage of CNN and RNN is to employ the CNN as a preprocessing operation for the RNN, i.e., to connect the RNN after the CNN. Therefore, as shown in Figure 1, two feature representations generated by the two parallel MCNN modules are concatenated into one feature representation as the input to feed into the two sequential GRU layers. In each GRU layer, the output dimension is set to 50, and the L2 regularization is set to 1 × 10${}^{-3}$.

#### 2.2.3. Prediction Module

A raw DNA sequence will be transformed into a feature matrix following the feature extraction layer. This feature matrix should be flattened into a vector and is then fed into a FCNN layer. The FCNN layer contains 256 neurons, and also uses the ReLU as the activation function, with L2 regularization set to 1 × 10${}^{-3}$ as well. The output layer exploits the sigmoid function to map the output of the FCNN layer to a classification probability ranging from [0, 1], which will be used as the final prediction result.

In the DeepNup model, a batch normalization layer is followed after each convolution layer to accelerate the training speed and avoid the disappearance of the gradient during the training process, and a dropout layer with a dropout value 0.5 is used after each max pooling layer and a GRU layer to prevent overfitting, whereas these two types of layers are not shown in Figure 1.

### 2.3. Model Training

In this work, the prediction of nucleosome positioning can be regarded as a binary classification problem, so the binary cross-entropy loss function is chosen to calculate the error, which is defined as follows:

$$Loss=-\frac{1}{N}\sum_{i=1}^{N}y_{i}\log\left(p\left(y_{i}\right)\right)+\left(1-y_{i}\right)\log\left(1-p\left(y_{i}\right)\right)$$

where *N* is the number of the labeled samples in the training set, $y_{i}\in \left\{0,1\right\}$ is the true label of the sample *i*, and $p(y_{i})$ is the predicted probability of the sample *i*. The DeepNup model is trained by using the Adam optimization algorithm with a batch size of the samples set to 64 and the learning rate set to 3 × 10${}^{-3}$ to update the model weights. To obtain a good generalization performance and prevent overfitting, the early stop mechanism is adopted, in which if the validation loss is no longer decreased after 15 epochs, then the training process will be interrupted. The DeepNup model is implemented by utilizing the Tensorflow2.5 framework, and it runs on a NVIDIA GeForce GTX 1080ti GPU.

## 3. Results and Discussion

In this section, to verify the effectiveness of the DeepNup method, we carried out a series of experiments on two to three datasets. First, we provided an introduction to the three datasets, then introduced the five evaluation indicators for quantifying the performance of DeepNup, and finally evaluated the prediction performance of DeepNup and compared it with that of iNuc-PseKNC and DLNN-5.

### 3.1. Datasets

There are two commonly used benchmark datasets for evaluating the nucleosome positioning prediction methods. The first dataset, renamed DatasetNup_1, is downloaded from the published paper by Guo et al [35]. The DatasetNup_1 covers three types of species, i.e., *H. sapiens*, *D. melanogaster*, *C. elegans*, each of which contains a number of nucleosome sequences and linker sequences. The nucleosome sequences are defined as positive samples, while the linker sequences are defined as negative samples. All nucleosome sequences and linker sequences in the DatasetNup_1 have a length of 147 bp. The number of positive samples and negative samples for each species is shown in Table 1.

The second dataset, renamed DatasetNup_2, is downloaded from a published paper by Amato et al. [39]. The DatasetNup_2 consists of *H. sapiens*, *D. melanogaster*, and *Yeast species* [30], but it is different from the DatasetNup_1, which consists of *H. sapiens*, and *D. melanogaster*, containing three classes of sequences, respectively, including the *largest chromosome* (*LC*), *promoter* (*PM*), and *5’UTR exon regions* (*5U*). Yeast contains two classes of sequences, including *whole genome* (*WG*) and *promoter* (*PM*). Each class of sequences is comprised of a number of nucleosome sequences and linker sequences, corresponding to positive samples and negative samples, respectively. As with the DatasetNup_1, all sequences in the DatasetNup_2 also have a length of 147 bp. The number of positive samples and negative samples for each class is shown in Table 2.

In addition to the above two datasets, we constructed another dataset on Homo Sapiens genome assembly Hg38 and denoted it by DatasetNup_3. Specifically, we first collected the start and end indexes of nuclesome sequences on chromosomes from the NucMap [47]. Then, we searched for nuclesome sequences with a length of 147 bp by referring to the Hg38 file according to their start and end indexes. Negative samples were also randomly selected from the same chromosome containing the positive samples. Moreover, we employed CDhit [48] to reduce the homology between DNA sequences, and the homology parameter was set to 0.8. Finally, we randomly selected 3134 positive samples of nucleosome sequences and 3137 negative samples of linker sequences, which is listed in Table 3.

### 3.2. Evaluation Metrics

In order to evaluate the classification performance of DeepNup and compare it with other nucleosome positioning methods, five evaluation metrics [30,35,41], including Sensitivity ($S_{n}$), Specificity ($S_{p}$), Accuracy (ACC), Matthews correlation coefficient (MCC), F1_Score, and the area under receiver operating characteristic curve (AUROC), are adopted in this work, in which the first five metrics are defined as follows:   

$$S_{n}=\frac{T_{p}}{T_{p}+F_{n}}$$

$$S_{P}=\frac{T_{n}}{T_{n}+F_{p}}$$

$$ACC=\frac{T_{p}+T_{n}}{T_{p}+F_{n}+T_{n}+F_{p}}$$

$$MCC=\frac{T_{p}\times T_{n}-F_{p}\times F_{n}}{\sqrt{\left(T_{n}+T_{n}\right)\times \left(T_{n}+F_{p}\right)\times \left(T_{p}+F_{n}\right)\times \left(T_{p}+F_{p}\right)}}$$

$$F1\_Score=\frac{2\times T_{P}}{2\times T_{p}+F_{p}+F_{n}}$$

where $T_{p}$ and $F_{p}$ denote the numbers of true and false positive samples, respectively, and $T_{n}$ and $F_{n}$ denote the numbers of true and false negative samples, respectively. The metric $S_{n}\in \left[0,1\right]$ denotes the rate of the correctly predicted positive samples to all positive samples (i.e., nucleosome sequences), with $S_{n}=1$, meaning that all nucleosome sequences are correctly identified, and with $S_{n}=0$, meaning that all nucleosome sequences are predicted to be linker sequences. The metric $S_{p}\in \left[0,1\right]$ denotes the rate of the correctly predicted negative samples to all negative samples (i.e., linker sequences), with $S_{p}=1$ meaning that all linker sequences are correctly identified, and with $S_{p}=0$ meaning that all linker sequences are predicted to be nucleosome sequences. The metric ACC∈[0,1] denotes the rate of both positive and negative samples that are correctly identified to all samples. The metric MCC∈[−1,1] is used to measure the prediction quality of a binary classifier, with MCC=1 meaning that the prediction is completely consistent with the truth, with MCC=0 representing a random prediction, and with MCC=−1 representing a completely opposite prediction. The metric F1_Score∈[0,1] combines both precision and recall.

The metric AUROC is often used to quantify the performance of a binary classifier, especially for imbalanced datasets. The AUROC is defined as the area under the receiver operating characteristic (ROC) curve, which is plotted by taking the metric $S_{n}$ as the *X*-axis and the metric $1-S_{p}$ as the *Y*-axis at different thresholds. The AUROC usually ranges from 0.5 to 1, with the closer the value of AUROC being to 1, the better the performance of the classifier.

### 3.3. Experimental Results

We performed experiments on the DatasetNup_1 and DatasetNup_2, and compared the prediction performance of DeepNup to that of several state-of-the-art methods. The prediction performance on the two datasets is discussed in detail, as follows.

#### 3.3.1. Prediction Performance on the DatasetNup_1

For the DatasetNup_1, a 10-fold cross validation scheme was utilized to evaluate the prediction performance of DeepNup. Specifically, the dataset for each species was randomly divided into 10 sub-datasets, which have an approximately equal size. According to the notion of 10-fold cross validation, one of the 10 sub-datasets was regarded as the test dataset, and the other 9 sub-datasets are integrated and regarded as the training dataset; so, 10 groups of training and test datasets were formed. Additionally, approximately 5% of each training dataset was randomly selected as the validation dataset. One model for nucleosome positioning prediction was trained and tested by using a group of training and test datasets, so 10 prediction models were obtained. In this work, the five evaluation metrics, i.e., $S_{n}$, $S_{p}$, ACC, MCC, F1_Score, AUROC, as defined above, were computed based on the test results of the corresponding models. The final value of each evaluation metric was obtained by averaging 10 values of the corresponding metric over the 10 test datasets.

**Figure 2:**
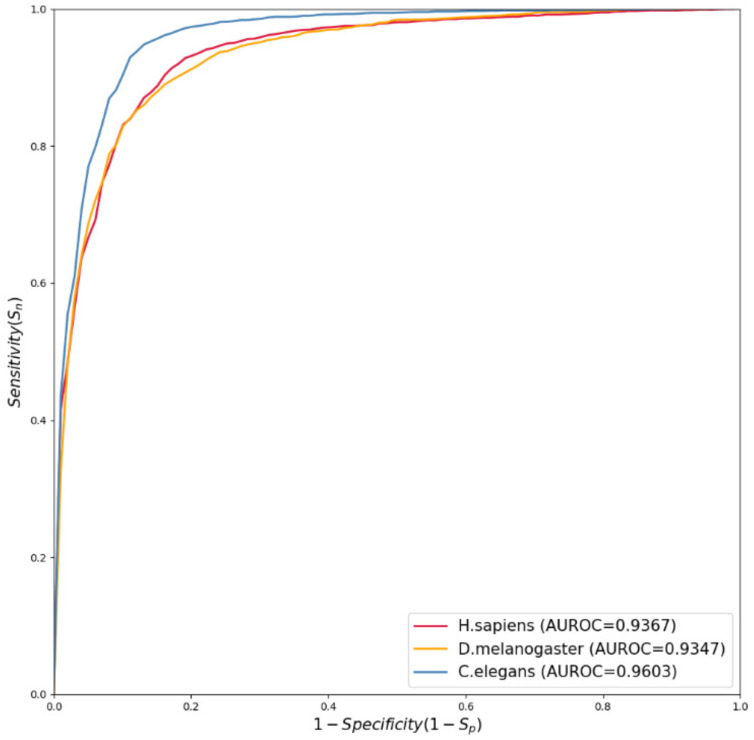
ROC curves plotted based on 10-fold cross validation test results across 3 species.

The classification performance measured by the six metrics $S_{n}$, $S_{p}$, ACC, MCC, F1_Score, and AUROC over three different datasets of *H. sapiens*, *D. melanogaster*, and *C. elegans* were listed in Table 4 for our proposed method. In addition, in order to obtain the optimal setting of *k* in the *k*-tuple nucleotide composition, we compared the results under the two cases k=3 and k=5, as shown in Table 4. It can be observed that DeepNup with k=3 achieved a better performance. Moreover, the ROC curves for *H. sapiens*, *D. melanogaster*, and *C. elegans* are shown in Figure 2, and the corresponding AUROC value for each ROC curve is 0.9367, 0.9347, and 0.9603, respectively.

Subsequently, we compared the prediction performance of DeepNup with that of two baseline methods, i.e., iNuc-PseKNC [35] and DLNN-5 [43]. iNuc-PseKNC was proposed based on SVM, and DLNN-5 is a DL-based method. The results of comparison between DeepNup and iNuc-PseKNC, DLNN-5 in three species were shown in Table 5. As seen from Table 5, for *H. sapiens*, DeepNup outperforms the other two baseline methods in terms of all the six metrics, and for *D. melanogaster* and *C. elegans*, DeepNup has a better performance than iNuc-PseKNC in terms of the five metrics, and than DLNN-5 in terms of $S_{p}$, ACC, MCC, and AUROC. In terms of the major metric AUROC, the values of AUROC are increased by 3.26% and 2.89% on average of three datasets, respectively. These results demonstrated that DeepNup is a powerful DL-based tool for nucleosome positioning identification.

#### 3.3.2. Prediction Performance on the DatasetNup_2

Because there is a large amount of noise in the DNA sequences of the eight datasets in the DatasetNup_2, it is a challenging task for DeepNup and other predictors to accurately identity nucleosome positioning for these datasets. For the DatasetNup_2, the 10-fold cross validation schema was also adopted to evaluate the performance of DeepNup. The results of the comparison between DeepNup and DLNN-5 on the eight datasets were shown in Table 6. As can be seen, DeepNup has a better performance than that of DLNN-5 in terms of ACC, MCC, and AUROC on all eight datasets. While in terms of $S_{p}$, $S_{n}$ and F1_Score, DeepNup outperforms DLNN-5 on most of the eight datasets. These results manifested that DeepNup is competitive in nucleosome positioning prediction.

#### 3.3.3. Prediction Performance on the DatasetNup_3

In order to verify the robustness of DeepNup, we conducted a comparative experiment on the DatasetNup_3, which was collected from Homo Sapiens genome assembly Hg38. Specifically, we also adopted the 10-fold cross validation schema to evaluate the performance of DeepNup and the baseline method DLNN_5. The comparative results of DeepNup with DLNN_5 were shown in Table 7. It can be observed that DeepNup outperforms DLNN_5 in terms of all metrics except $S_{p}$, which further demonstrated the strong robustness of our proposed method.

## 4. Conclusions

In this work, we proposed a new method, namely DeepNup, for predicting nucleosome positioning only from DNA sequences. To facilitate DeepNup to derive more useful features from DNA sequences, a hybrid feature encoding scheme consisting of One-hot encoding and TNC encoding is input to DeepNup in parallel. To extract features of DNA sequences in different scales, a MCNN module consisting of two parallel convolution kernels with different sizes is designed, wherein the convolution kernels with relatively small receptive fields are used to extract more detailed local features of DNA sequences, while the convolution kernels with relatively large receptive fields are used to extract more abstract local features of DNA sequences. To capture long-range dependencies of DNA sequences, a GRU module consisting of two sequential GRU layers is designed. To verify the effectiveness of DeepNup, a series of experiments were performed on two benchmark datasets by utilizing 10-fold cross validation. More specifically, compared to two baseline methods, DeepNup exhibited a competitive prediction performance in identifying nucleosome positioning on the two benchmark datasets, especially in terms of the three major performance metrics ACC, MCC, and AUROC.

According to the results on the DatasetNup_2, DeepNup has a relatively worse performance in predicting nucleosome positioning on this dataset, because the DNA sequences in this dataset contain much noise. As a result, it is worthwhile to develop novel DL-based methods by adopting new DL techniques to improve the performance on the DatasetNup_2 for nucleosome positioning prediction.

The variable nature of DNA sequences in real application scenarios remains a challenge for DL-based methods to identify nucleosome positioning from raw DNA sequences. Nevertheless, our method and existing DL-based methods are species-specific trained models, so these methods suffered from poor generalization for cross-species validation. Bert [49] has been demonstrated to be an effective method for constructing general models. Consequently, it is interesting and promising to employ Bert to construct a general model for nucleosome positioning prediction across different species, More specifically, the BERT-based model was first trained on the whole data of all species as the pretrained models, and then the pretrained model was fine-tuned on the training data of the target species. Finally the fine-tuned model was evaluated on the test data of the target species.

## Figures and Tables

**Table 1:** The number of positive samples and negative samples in the DatasetNup_1.

	*H. sapiens*	*D. melanogaster*	*C. elegans*
Nucleosome sequences	2273	2900	2567
Linker sequences	2300	2850	2608
Total	4573	5750	5157

**Table 2:** The number of positive samples and negative samples in the DatasetNup_2.

	*H. sapiens*			*D. melanogaster*			*C. elegans*	
	*LC*	*PM*	*5U*	*LC*	*PM*	*5U*	*WG*	*PM*
Nucleosome sequences	97,209	56,404	11,769	46,054	48,251	4669	39,661	27,373
Linker sequences	65,563	44,639	4880	30,458	28,763	2704	4824	4463
Total	162,772	101,043	16,649	76,512	77,014	7373	44,485	31,836

**Table 3:** The numbers of positive samples and negative samples in the DatasetNup_3.

	*Nucleosome sequences*	*Linker sequences*	*Total*
*H. sapiens*	3134	3137	6271

**Table 4:** Prediction performance of DeepNup in terms of 6 metrics via 10-fold cross validation for 3 species.

	$S_{n}$	$S_{p}$	ACC	MCC	F1_Score	AUROC
*H. sapiens*						
** k=3 **	0.8896	**0.8560**	**0.8705**	**0.7421**	**0.8722**	**0.9367**
** k=5 **	0.9038	0.8359	0.8657	0.7358	0.8711	0.9343
*D. melanogaster*						
** k=3 **	**0.8659**	**0.8598**	**0.8621**	**0.7249**	**0.8638**	**0.9347**
** k=5 **	0.8406	0.8508	0.8454	0.6911	0.8459	0.9210
*C. elegans*						
** k=3 **	0.9267	**0.8910**	**0.9076**	**0.8166**	**0.9088**	**0.9603**
** k=5 **	**0.9318**	0.8740	0.9009	0.8041	0.9035	0.9598

*Note*: The best results are indicated in bold.

**Table 5:** Comparison of DeepNup with iNuc_PseKNC and DLNN_5 via 10-fold cross validation in 3 species.

Species	Metrics	iNuc_PseKNC	DLNN_5	DeepNup
*H. sapiens*	$S_{n}$	0.8786	0.8834	**0.8869**
	$S_{p}$	0.8470	0.8229	**0.8560**
	ACC	0.8627	0.8537	**0.8705**
	MCC	0.73	0.7026	**0.7421**
	F1_Score	-	0.8545	**0.8722**
	AUROC	0.925	0.8560	**0.9367**
*D. melanogaster*	$S_{n}$	0.7831	**0.8781**	0.8659
	$S_{p}$	0.8165	0.8333	**0.8598**
	ACC	0.7997	0.8560	**0.8621**
	MCC	0.60	0.7206	**0.7249**
	F1_Score	-	**0.8656**	0.8638
	AUROC	0.874	0.9316	**0.9347**
*C. elegans*	$S_{n}$	0.9030	**0.9304**	0.9267
	$S_{p}$	0.8355	0.8634	**0.8910**
	ACC	0.8690	0.8962	**0.9076**
	MCC	0.74	0.8033	**0.8166**
	F1_Score	-	0.903	**0.9088**
	AUROC	0.935	0.9573	**0.9603**

*Note*: The best results are indicated in bold. The values of *MCC*, *F1_Score*, *AUROC* in DLNN-5 were computed based on its source code.

**Table 6:** Comparison of DeepNup with DLNN_5 via 10-fold cross validation on 8 datasets.

	$S_{n}$	$S_{p}$	ACC	MCC	F1_Score	AUROC
*H. sapiens_LC*						
**DLNN_5**	**0.9304**	0.8504	0.8743	0.7351	0.9061	0.9126
**DeepNup**	0.9273	**0.8556**	**0.8773**	**0.741**	**0.9065**	**0.920**
*H. sapiens_PM*						
**DLNN_5**	**0.8955**	0.7818	0.8192	0.6409	0.8556	0.8664
**DeepNup**	0.8939	**0.7914**	**0.826**	**0.6531**	**0.8574**	**0.8758**
*H. sapiens_5U*						
**DLNN_5**	**0.8173**	0.7931	0.7962	0.4667	0.8698	0.768
**DeepNup**	0.8092	**0.8033**	**0.8032**	**0.4887**	**0.8729**	**0.790**
*D. melanogaster_LC*						
**DLNN_5**	**0.7451**	0.7092	0.7175	0.3901	**0.7931**	0.7233
**DeepNup**	0.7395	**0.710**	**0.7189**	**0.3922**	0.7920	**0.7246**
*D. melanogaster_PM*						
**DLNN_5**	0.7670	0.7054	0.7421	0.4277	0.8170	0.7407
**DeepNup**	**0.7692**	**0.7373**	**0.7441**	**0.4326**	**0.8182**	**0.7437**
*D. melanogaster_5U*						
**DLNN_5**	0.7110	**0.7173**	0.7158	0.352	0.8024	0.690
**DeepNup**	**0.7274**	0.7151	**0.7169**	**0.356**	**0.8044**	**0.7028**
*Yeast_WG*						
**DLNN_5**	**0.8078**	0.9484	0.9375	0.6403	0.9656	0.9343
**DeepNup**	0.7919	**0.9564**	**0.9420**	**0.6768**	**0.9610**	**0.9430**
*Yeast_PM*						
**DLNN_5**	0.8115	0.9384	0.9247	0.661	0.9571	0.9322
**DeepNup**	**0.8131**	**0.9467**	**0.9311**	**0.6958**	**0.9678**	**0.9410**

*Note*: The best results are indicated in bold. The values of all 6 metrics in DLNN-5 are computed via 10-fold cross validation based on the source code of DLNN-5.

**Table 7:** Comparison of DeepNup with DLNN_5 via 10-fold cross validation on the DatasetNup_3.

Model	$S_{n}$	$S_{p}$	ACC	MCC	F1_Score	AUROC
**DeepNup**	**0.9414**	0.9443	**0.9424**	**0.8853**	**0.9422**	**0.9845**
**DLNN_5**	0.9193	**0.9476**	0.9325	0.8660	0.9313	0.9814

*Note*: The best results are indicated in bold.

## Data Availability

Not applicable.
